# Evaluating a targeted person-centred pain management intervention programme in lumbar spine surgery - a controlled segment-specific before-and-after interventional design

**DOI:** 10.1186/s12913-024-10769-8

**Published:** 2024-03-08

**Authors:** Eva Angelini, Axel Wolf, Helle Wijk, Helena Brisby, Adad Baranto

**Affiliations:** 1 Dept of Orthopaedics, Institute of Clinical Sciences, at Sahlgrenska Academy, University of Gothenburg, Gothenburg, Sweden; 2 Institute of Health and Care Sciences, at Sahlgrenska Academy, University of Gothenburg, Gothenburg, Sweden; 3 Institute of Nursing and Health Promotion, Oslo Metropolitan University, Oslo, Norway; 4 Dept of Anaesthesia, Operation & Intensive Care, Sahlgrenska University Hospital, Gothenburg, Sweden; 5 Dept. of Quality Improvement, Sahlgrenska University Hospital, Gothenburg, Sweden; 6 Architecture, Chalmers University of Technology, Gothenburg, Sweden; 7 Dept. of Orthopaedics, Sahlgrenska University Hospital, Gothenburg, Sweden

**Keywords:** Person-centred care, Postoperative pain management, Co-creation, Shared decision-making, Patient satisfaction, Spine surgery

## Abstract

**Background:**

Postoperative pain management in lumbar spine surgery care remains a challenge. The aim of this study was to evaluate the impact of a person-centred postoperative pain management intervention programme on lumbar spine surgery patients on postoperative pain, shared decision-making, and satisfaction with postoperative pain management.

**Methods:**

The study was performed with a controlled before-and-after interventional design in an orthopaedic unit at a university hospital. Person-centred pain management for patients undergoing spine surgery was developed in co-creation by a multi-professional team and implemented throughout the care pathway. The *usual care* group (pre-intervention) served as a comparison to the *intervention* group. Pain intensity, shared decision-making in pain management, and patient satisfaction with results of pain management, served as patient-reported measures, collected using the International Pain Outcomes questionnaire and analysed using descriptive statistics.

**Results:**

The intervention showed no benefit for patients’ pain and satisfaction, while shared decision-making in pain management was significant lower in the *intervention* group than in the conventional group. The per-protocol analysis showed no significant differences between groups.

**Conclusion:**

The initial assumption of the study, that the implementation of a co-created structured person-centred care pathway would improve patient-reported outcomes, was not confirmed. The periodically low fidelity to the intervention due to organizational constraints (due to sub-optimal organizational conditions and managerial support) may have affected the results.

**Supplementary Information:**

The online version contains supplementary material available at 10.1186/s12913-024-10769-8.

## Introduction

Every year, approximately 300 million surgeries are conducted globally [1], with many patients reporting dissatisfaction with pain management [2]. Despite advances in techniques and pharmaceuticals, postoperative pain management continues to be a challenge in clinical care [3–5]. In orthopaedic spine surgery care, pain and pain management are crucial aspects in improving outcomes. Pain following surgery has multiple simultaneous physical causes, causing great suffering for patients. Several factors influence how individuals experience and cope with the pain after spine surgery [6]. It has been shown that more than half of patients experienced inadequately managed pain within the 24 h following their surgical spine procedures [7], with a considerable number developing chronic pain, possibly stemming from inadequately managed acute postoperative pain [8].

One way of approaching postoperative pain in health care involves shared decision-making in pain management as a part of person-centred care (PCC). PCC has emerged as a vital component of sustainable, high-quality health care [9, 10]. While there is currently no universally accepted definition of PCC [11], it is broadly acknowledged that its core focus is on treating the patient as an individual person, rather than solely addressing the disease [12]. The Gothenburg Centre for Person-Centred Care (GPCC) has developed and tested a specific approach to PCC. In the GPCC model, Ekman et al. in 2011 [12] elaborate three parts of person-centredness. The patient’s personal narrative serves as the starting point, forming the foundation for a collaborative relationship between the patient and health-care professionals. This partnership is formalized, documented, and sustained through a jointly crafted GPCC plan. A central goal of the GPCC approach is to involve and empower patients as proactive collaborators in their healthcare through principles of shared decision-making [12].

A structured PCC pathway for patients during orthopaedic hip surgery produced significant reductions in length of hospital stay in clinical controlled studies, as well as decreases in pain and increases in patients’ activities of daily life [13, 14]. In studies, Angelini et al. [15, 16] showed that lack of coherence in pain management created a risk to patients of inadequate pain management. This challenge has prompted efforts to redesign pain-management care around the needs of the patient. However, introducing change in complex health care organizations is not easy, not least as health care is already in a continuous state of change to meet demands for improved quality and safety [3, 10, 17]. Implementing PCC in health care is therefore likewise challenging, as exemplified by Ekman et al.’s discovery of a fidelity of only 60% to the intervention in chronic heart failure care when implementing PCC, despite a rigorous design [18]. Implementation fidelity refers to the extent to which interventions are carried out according to the original intentions or designs established by their developers or designers, identified by evaluating adherence in implementation [19]. Moore et al. [20] identified factors acting as barriers to PCC interventions, such as health-care professionals’ attitudes and their keeping to traditional practices and structures, as well as limited time for training before implementation of a PCC intervention. They also identified involvement of stakeholders and key persons as enabling the delivery of PCC [20]. It has been argued that interventions in complex health-care settings need to distinguish between the clinical intervention and its implementation [21, 22]. Therefore, the current study will report on both patient-reported outcomes (PROs) and fidelity to the intervention during its implementation.

The study aims to evaluate the impact of a person-centred pain management intervention programme on lumbar spine surgery patients on postoperative pain, shared decision-making, and satisfaction with postoperative pain management. The assumption was that the implementation of a co-created structured PCC pathway would improve PROs.

## Methods

### Study design

The current study has a controlled before-and-after interventional design. The *usual care* group (control) data were collected from March 2017 to February 2018; the intervention started in October 2018, data for the *intervention* group (experimental) being collected from April 2019 to March 2020. The present study evaluates the impact of the intervention on patient-reported experiences and outcomes with the validated International Pain Outcomes (IPO) questionnaire [23].

### Setting

In Sweden, health care is predominantly funded under the Beveridge structure, which is characterized by state funding and a taxation-based system. This study was conducted in a care unit specializing in spine surgery at a university hospital in Sweden. The care unit comprises an out-patient clinic where patients have their pre- and postoperative consultations, and an orthopaedic surgery ward. In the ward, besides patients undergoing elective spine surgery, a few beds are used for trauma patients and orthopaedic oncology patients, including both children and adults. Through the study period, the number of beds in the ward varied between 19 and 28. The ward has approximately 1300–1400 care episodes per year. The staff comprise physicians, registered nurses (RNs), assistant nurses (ANs), physiotherapists (PTs), assistant PTs, and administrative personnel.

### The intervention

Subsequent to the enrolment of the *usual care* group, a multi-professional expert group was formed to design and facilitate the PCC intervention. It comprised first-line management, orthopaedic surgeons, assistant doctors, RNs, PTs and ANs. A total of nine professionals was active in the group simultaneously. The assistant doctors rotated as they worked for approximately six months in the unit; likewise, RNs altered due to staff turnover. The expert group was tasked with improving postoperative pain management care: it started by mapping usual care and subsequently co-created the intervention. The mapping of the unit’s usual care procedures revealed the following. Documentation in patient records lacked cohesiveness, making it challenging to effectively track and manage patients’ reported pain and pain management. Daily ward rounds were perceived as stressful and regarded as inadequate and inefficient by health-care staff. These rounds disrupted patient care due to their unpredictable timing, and decision-making was prolonged because assistant doctors were often left unadvised while orthopaedic surgeons were either in the operating theatre or attending to outpatient clinic duties. In consequence, this resulted in patients not receiving timely care. Upon discharge, there was no established protocol for providing patients with written information regarding the care received during their hospital stay, nor instructions for post-discharge care. The group met 10 times during 2018 and additional meetings were held within each professional group (1–2 times) to establish dissemination strategies for each profession. The rationale for the intervention and the patient perspective came from patient interviews performed before the intervention, which highlighted the absence of distinct structures concerning patients’ pain and pain management during and after elective lumbar spine surgery [15, 16]. The intervention as finally co-created comprised multiple strategies focusing on patients’ postoperative pain management, consisting of person-centred structures (see Table 1). Further, a care plan was created to enable coherent postoperative pain management by gathering all documentation regarding pain in one place in patients’ records and maintaining and updating patients’ narratives throughout hospitalization (Table 1).

**Table 1:** The intervention: an overview of the intervention of person-centred structures

Structural change	Explanation of change process
RN admission interview with the patient about their pain (Start February 2019)	Pre-intervention, two workshops (WS) on PCC with RNs in the outpatient clinic. Subsequently, RNs employed after WSs received information and training as part of their general training. The novel routine: The RN obtained the patient’s narrative during the pre-admission visit. The narrative was documented in a care plan. A tentative PCC plan was written by the RN. The care plan was finalized and updated with the patient at admission to the ward.
Care plan with focus on pain and pain management (Start February 2019)	A flowchart was developed by RNs in the expert group. Information about the use of the care plan was communicated to all staff working in close patient care. The novel routine: Continuous documentation of pain and pain management in the care plan.
Ward round routine with explicit roles (Start October 2018)	The routine was established by consensus in the expert group and then agreed to by relevant first-line managers. The novel routine: Checklist and timing for the ward round. All professions represented in the ward round. Physician leading the round; RN summarizing pain issues addressed in the care plan.
Written patient discharge summary (Start November 2018)	Routines for templates for different surgical interventions were established by a group of physicians. The novel routine: Ward medical secretaries were assigned to enter the template in patients’ records. At discharge, the physician adjusts the summary for each patient.

A more detailed explanation of the intervention is provided by Angelini et al. [24].

### Participants and recruitment

The study population comprised all adult patients admitted to the ward from the surgery waiting list to undergo elective lumbar spine surgery as their main procedure. The estimated sample size of 128 patients in total was calculated in a statistical power analysis further explained in the statistics section below. In the given scenario, a strategic approach was employed, enrolling 275 patients to allow for the anticipated high attrition rates. This pre-emptive measure was particularly pertinent, given the considerable operational stress experienced by the health-care organization. The unexpected and complete relocation of surgical units during the intervention period, an element not accounted for in the original study design, significantly impacted the study’s execution. Preoperative exclusion criteria were malignancy, rheumatic disorder, stroke, deformities of the thoracolumbar spine (e.g., idiopathic scoliosis), a planned hospital stay shorter than 24 h and/or insufficient Swedish language fluency. Postoperative exclusion criteria were complications necessitating re-operation.

Consecutive sampling was used both in the *usual care* and *intervention* groups. Eligible patients were invited to participate during the pre-surgery visit, while, for the *usual care* group, this invitation could also be given by an RN at admission to the ward. Participants were informed that they were either part of the pre-intervention (i.e., *usual care*) group (March 2017 to February 2018) and treated according to the pre-existing routines in the unit, or part of the *intervention* group (April 2019 to March 2020) and treated according to the novel routines (Table 1). Patients received information about the study and those willing to participate were guided through the study protocol. All participants gave their informed consent in writing.

### Data collection

Patients completed the first set of questions in the International Pain Outcomes (IPO) questionnaire [23] on the first day after surgery while still in hospital. At the one-month follow-up, the questionnaires were sent to the patients’ homes. If not returned, one phone call was made and/or one reminder letter was sent to patients. Questionnaire answers at each time point were to be based on patients’ experiences during the previous 24 h.

Demographic data for the groups include age, gender, type of surgery, and the preoperative risk assessment, according to the American Society of Anesthesiologists (ASA) physical status classification system [25] (Table 2). The ASA classification served as a proxy for understanding patients’ conditions, it being used to classify perioperative risk and to control for the potential changes in case severity between the *usual care* and *intervention* groups.

### Intervention outcomes

#### Patient-reported outcomes (PROs)

PROs were scored by the patient in the IPO questionnaire [23]. This depicts the numerous barriers to provision of adequate postoperative pain management. Patients rate their perceived postoperative pain and parameters related to pain on 13 items (mainly based on 11-point Likert scales), of which three were used here. A Swedish version of the questionnaire is available and permission to use it was given by the PAIN OUT registry. In the validation of the IPO questionnaire, the psychometric quality was judged satisfactory [26].

The relevant PROs scored were pain intensity: Numerical Rating Scale (NRS) (0 (no pain) to 10 (worst possible pain)); perceptions of care: shared decision-making in pain management (0 (not at all) to 10 (very much so)); patient satisfaction with results of pain management (0 (extremely dissatisfied) to 10 (extremely satisfied)).

#### Fidelity to the intervention

To evaluate compliance with the intervention and gather data for a per-protocol analysis, we established organizational outcomes. These outcomes specifically concentrated on parameters related to adherence, such as the frequency of approved utilization of the care plan, and the completion of a written patient discharge summary (evaluated through patient record review).

The care plan was considered approved if the care plan template had been adjusted to the specific patient, and if documentation on pain was collected in the established care plan with focus on pain and pain management in the patient record.

The written patient discharge summary template, corresponding to the patient’s surgical intervention and postoperative care, was considered approved if it had been adjusted to the specific patient. A patient record review was conducted to see how well the agreed intervention plan was performed for each patient (author EA).

### Statistical analysis

Data were described in terms of means (standard deviations (SDs)) for continuous variables and frequencies (percentages) for categorical variables. Variables, checked for normality by visual inspection of histograms, were judged to be approximately normally distributed. For comparison of continuous variables, Student’s independent *t*-test was used, with mean differences and 95% confidence intervals (CIs); Pearson’s chi-squared test was performed to evaluate associations between categorical variables. All tests were two-sided and conducted with alpha set at 0.05. The data were analysed using IBM SPSS Statistics for Windows, version 27.0. Armonk, NY: IBM Corp.

Statistical power: Primary outcome measure för the current study was: “Satisfaction with postoperative pain management”, compared between Groups 1 and 2 assessed on a 0–10 scale (0 = not satisfied at all; 10 = very satisfied). Clinical relevance was agreed as a difference of 1 point, this being deemed a clinically important difference by the research group. This yields an estimated sample size of 128 patients in total, 64 per group, based on a t-test assuming SD1 = SD2 = SD.

Null hypothesis: Satisfaction in Group 1 = Satisfaction in Group 2. Alternative hypothesis: Satisfaction in Group 2 (*intervention*) is not equal to satisfaction in Group 1 (*usual care*).

To mitigate potential variability in clinical pathways and outcomes stemming from the utilization of two surgical approaches (fusion vs. non-fusion), we conducted a subgroup analysis. This analysis categorized patients into non-fusion and fusion groups based on the severity of surgical intervention and clinical understanding of the postoperative recovery period for each respective group This subgroup analysis was crucial for ensuring that the differential effects of the surgical methods were adequately understood and accounted for in the study’s findings.

## Results

**Fig. 1:**
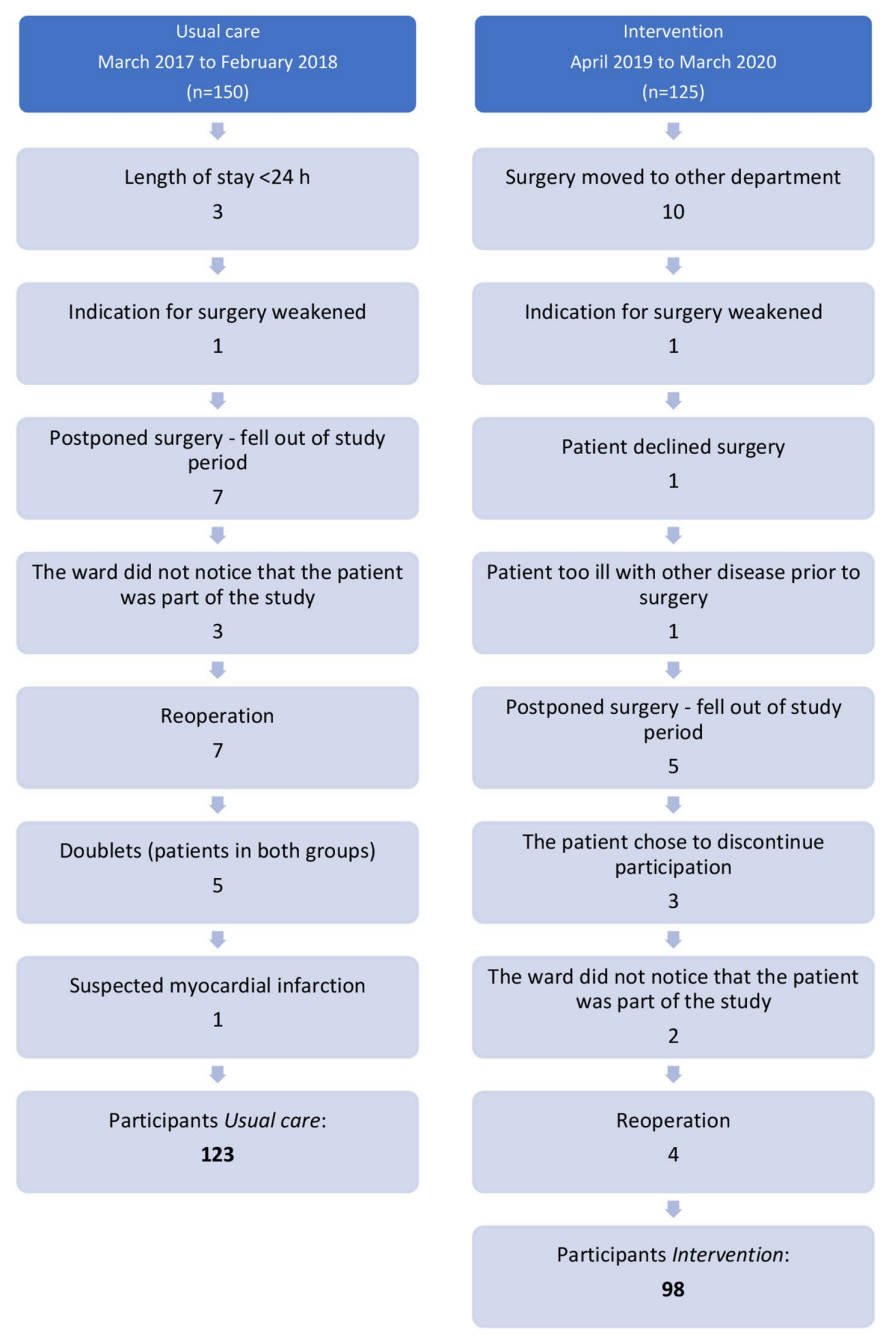
Flow-chart of drop-outs in the *usual care* and *intervention* groups

In total, 275 consecutive patients were invited to participate (*usual care* = 150; *intervention* = 125); however, after applying exclusion criteria, the final number of included participants was 221 (*usual care* = 123; *intervention* = 98). Most exclusions (*usual care* 27 (18%); *intervention* 27 (22%)) were for organizational reasons or postoperative complications (Fig. 1). A comparison of the patients allocated to the *intervention* group with the *usual care* group was made. In addition, a per-protocol analysis of the participants who received the complete intervention was performed and compared to the *usual care* group.

### Comparison between participants in the ***intervention*** and usual care groups

The groups were similar in distribution of gender and preoperative (baseline) pain levels. The *intervention* group was significantly older (*p* = 0.007). There were no differences in ASA class distributions between the *usual care* and *intervention* groups (for details see Table 2).

**Table 2:** Demographics: *usual care* and *intervention* groups

Variable	Usual care group *n = 123)*	Intervention group *(n = 98)*	***p***
***Female, n (%)***	62 (50.4)	54 (55.1)	0.49
***Age, years, mean (SD)***	60 (15.8)	65 (12.4)	**0.007**
***ASA I-II n (%)***	87 (71)	61 (62)	0.18
***ASA III n (%)***	36 (29)	37 (37)	
***Preoperative pain, mean (SD)***	6.7 (2.3)	7.1 (2.0)	0.058

### Fidelity towards the intervention (preoperative admission interview, care plan and discharge summary)

In the *usual care* group, none of the subsequently co-created measures was used in treatment.

*Intervention* group:

- Preoperative admission interview documented in care plan in the patient record: 100%.
- Care plan approved according to criteria: 72%.
- Written patient discharge summary approved according to criteria: 43%.

### Patient-reported outcomes

#### Pain intensity

Pain intensity (NRS for 24 h) at Day 1 and one-month follow-up showed no statistically significant differences between the groups (Table 3).

#### Shared decision-making in pain management decisions and patient satisfaction with results of pain management

At the one-month follow-up, there was a statistically significant difference about shared decision-making in pain management decisions in favour of *usual care* group (*n* = 98) compared with the *intervention* group (*n* = 75; *p* = 0.024): there was no statistically significant difference in satisfaction with the result of pain management between the groups (Table 3).

**Table 3:** Day 1 and one-month follow-up: pain intensity. One-month follow-up: shared decision-making in pain treatment and satisfaction with the result of pain management

	Follow-up	Cross-section	n	Mean NRS	SD	***p*** Usual care vs. Intervention
***Pain intensity***	Day 1	Usual care	99	6.1	2.6	0.313
		Intervention	66	6.5	2.7	
	Month 1	Usual care	101	5.2	2.8	0.705
		Intervention	77	5.4	2.9	
***Shared decision-making***	Month 1	Usual care	98	5.2	3.7	**0.024**
		Intervention	75	3.9	3.7	
***Satisfaction***	Month 1	Usual care	100	7.4	2.8	0.150
		Intervention	78	6.7	2.8	

In the subgroup analysis the *intervention* group exhibited a higher percentage of patients undergoing fusion as a surgical procedure (*p* = 0.002) (supplementary file, table S1). Additionally, there was a disparity in ASA class distributions between the *usual care* and *intervention* groups, with the non-fusion group showing a significantly greater proportion of ASA III (*p* = 0.013) (supplementary file, table S1). In terms of shared decision-making, fusion patients in the *intervention* group (*n* = 36) reported a significantly lower level than those in the *usual care* group (*n* = 30; *p* = 0.05) (supplementary file, tables S2). However, no other statistically significant differences were observed in the subgroup analysis for either pain intensity or satisfaction with the outcome of pain management (supplementary file, tables S2, S3).

### Per-protocol analysis

#### Comparison between ***intervention*** group receiving the full PCC intervention and the ***usual care*** group 

Of 98 participants in the *intervention* group, the patient record review identified 31 as having received the entire PCC intervention (as described in Table 1): a per-protocol analysis was conducted on this group (*n* = 31) in comparison with the *usual care* group (*n* = 123) to illustrate the impact of the full PCC intervention (Table 1). The per-protocol group were similar to the *usual care* group in distribution of gender, ASA level, and preoperative (baseline) pain levels. However, the per-protocol *intervention* group had a higher mean age (*p* = 0.040).

#### Patient-reported outcomes

There was no statistically significant difference for pain intensity, shared decision-making, or satisfaction with the result of pain management, between the *usual care* group and the per-protocol group at Day 1 nor at the one-month follow-up. The difference in shared decision-making identified in the full group analysis (Table 3) was not seen in this sub-group analysis (*p* = 0.82).

## Discussion

The findings of our study revealed no discernible differences between the *intervention* and *usual care* (control) groups for patients’ reported pain levels and overall satisfaction with pain management outcomes. However, noteworthy distinctions emerged in the realm of shared decision-making in pain management, with the *intervention* group reporting lower scores than the *usual care* group. Upon conducting a per-protocol analysis, no statistically significant differences regarding shared decision-making in pain management were observed between the groups. Consequently, the initial hypothesis in our study, positing that the implementation of a co-created structured person-centred care pathway would yield improvements in patient-reported outcomes, was not substantiated by our empirical findings.

The findings suggest that adhering to a specific task (pain management) and using only some of the cornerstones in PCC is sub-optimal. Studies in PCC have shown that a systematic approach with structures involving the patients throughout the entire care continuum has the greatest effect [27]. As shown by Ekman et al. [18], the largest and most significant effect of a PCC intervention in chronic heart failure patients was observed when the intervention was continuously used throughout the care process [18]. In the present study, the results of the medical chart review clearly demonstrates that PCC was not implemented as a “comprehensive” strategy during the intervention, as with Ekman et al. [18] who likewise did not achieve fidelity in part of their intervention with patients with chronic heart failure in the ITT analysis [18]. However, in contrast to the Ekman et al. study [18], which used all components of the Gothenburg Framework of PCC, the current study only used those parts that suited the pain management context. The lack of a comprehensive PCC strategy in the current study may have influenced the results observed for shared decision-making in pain management. This indicates that PCC should be implemented as a “complete” strategy, comprising the ethics of person-centredness, as well as a systematic and structural approach involving the entire care chain (including all staff as well as the patient). Further, the negative result for shared decision-making could also be due to patients having increased expectations for PCC and, when not rigorously implemented, a reduced perception of participation in decision-making. Hence, if the patient expects shared decision-making but does not experience it, or the expected outcome of the pain management discussed is not achieved, satisfaction levels may very well be worse than if the patient were not part of the discussion in the first place (expectation vs. outcomes). Once again, the comprehensive PCC strategy is more than the sum of its individual parts.

Patient satisfaction is frequently utilized as a key metric to evaluate the quality of care in various clinical trials [28]. In our study, despite notable demographic differences such as age, medical complexity (as indicated by higher ASA scores), and the extent of surgical procedures undergone (as evidenced in subgroup analysis), satisfaction with pain management remained consistent. There exists disparity in research findings regarding the correlation between age and satisfaction with care. Studies have shown that older patients tend to report higher levels of satisfaction with their surgical experiences compared to younger patients [29–32]. Additionally, older individuals are more likely to express satisfaction with perceived shared decision-making during preoperative consultations [33]. However, some studies have failed to establish a significant correlation between age and patient satisfaction in surgical settings [34, 35]. In the postoperative period, improved patient satisfaction is linked to effective pain management [28, 36], involvement in decision-making, and being treated with respect and dignity [28, 33, 37]. Again, the *intervention* group was significantly older and therefore a possible explanation to the persistent levels of satisfaction with pain management.

Most individuals desire active participation in their health care, and engaging in shared decision-making has the potential to enhance patient satisfaction [33, 38, 39]. However, in this study shared decision-making decreased without affecting patient satisfaction with the result of pain management. Could it be that shared decision-making in pain management lends itself to tangible measurement at the time of the actual pain management decision, as opposed to satisfaction with pain management, which represents a more comprehensive and abstract construct over time.

Angelini et al. [24] describe the study site in the present study experiencing severe organizational strain during the implementation of the intervention [24]. Despite the study’s rigorous design, including co-creation by a multi-professional expert group allowing a close adaptation to the context, the unit could not avoid the effect of multiple external influences, such as relocation to other premises, high RN turnover, and the demand for increased productivity during the change process. Moreover, there was an increased level of co-morbidity in the patient group, as seen in higher ASA classifications (subgroup analysis). These internal and external organizational changes may have influenced the impact of the intervention through the interference of these changes with other routines and activities at the time of the implementation of the PCC pain management intervention, and due to such factors as less experienced staff and/or changes in patient-related factors.

### Strengths and limitations

A strength in the present study is that the investigation of fidelity, i.e. how well the intervention was applied, adds scrutiny and transparency to the implementation. An assessment of fidelity to an intervention is crucial when discussing findings which may influence future health care practice.

There are several limitations of the present study.

The impact of systematic change was reduced due to lack of longevity. This intervention was sensitive to external organizational influences. Though organizational strain is always present in health care organizations, the extent to which it was experienced during this study may have impacted the results. In addition, the before-and-after design implies a lengthy gathering of data problematic in an ever-changing and complex setting such as health care compared to, for example, a parallel RCT which was not deemed feasible because of the high risk of “contamination” (clinicians treating both groups) and because the change was made in just one clinic. The impact of systematic change was reduced due to lack of longevity. This intervention was sensitive to external organizational influences. That person-centred care was only partially implemented, instead of systematically encompassing the entire care process/continuum, may have also affected the outcomes. With hindsight, it was challenging to take only some parts of a PCC into a study of a complex setting and patient group. Further, the smallness of the sample of the *intervention* group receiving the full intervention (the per-protocol group) could only give an indication of the potential results of such interventions.

## Conclusions

Overall, no benefit evaluated with PROs could be seen from using a PCC intervention pain programme in a spine surgery unit using the present study set-up. Studying interventions in dynamic health organizations is challenging. The complex, ever-changing context may have influenced our study results. Future research in this area should acknowledge the profound impact of health care organizational complexity. Our results endorse the ongoing consideration of critical parameters, specifically pain and pain management, in perioperative care and recovery. Integrating this approach with Person-Centred Care has the potential to maintain care quality, even in health-care organizations facing challenges.

### Electronic supplementary material

Below is the link to the electronic supplementary material.

Supplementary Material 1

## Data Availability

The datasets generated and/or analysed during the current study are not publicly available due to the risk this poses to the confidentiality of participants. However, anonymised data are available from the corresponding author on reasonable request.

## Abbreviations

AN: assistant nurse
ASA: American Society of Anesthesiologists
IPO: International Pain Outcomes questionnaire
PRO: patient-reported outcomes
NRS: Numerical Rating Scale
PCC: person-centred care
PT: physiotherapist
RN: registered nurse
