# Supplementary material for: Evaluating a targeted person-centred pain management intervention programme in lumbar spine surgery - a controlled segment-specific before-and-after interventional design
Source: BMC Health Serv Res. 2024 Mar 8;24:315. doi: 10.1186/s12913-024-10769-8 (PMC10921751; doi:10.1186/s12913-024-10769-8)
Supplement: Supplementary file 1 — Supplementary Material 1 [file 12913_2024_10769_MOESM1_ESM.docx]

Table S1. Demographics: *Usual care* group and *intervention group*

| **Variable** | **Usual care group**  **(n = 123)** | **Intervention group**  **(n = 98)** | ***p*** |
| --- | --- | --- | --- |
| Female, n (%) | 62 (50.4) | 54 (55.1) | 0.49 |
| Age, years, mean (SD) | 60 (15.8) | 65 (12.4) | **0.007** |
|  |  |  |  |
| Surgical intervention, n (%) |  |  |  |
| Fusion | 36 (29.3) | 49 (50.0) | **0.002** |
| Non-fusion | 87 (70.7) | 49 (50.0) |  |
| Patients’ pre-hospitalisation  conditions (ASA), n (%) |  |  |  |
| Fusion |  |  |  |
| ASA I-II | 29 (81) | 39 (80) | 0.91 |
| ASA III | 7 (19) | 10 (20) |  |
| Non-fusion |  |  |  |
| ASA I-II | 58 (67) | 22 (45) |  |
| ASA III | 29 (33) | 27 (55) | **0.013** |
| Preoperative pain, mean (SD) |  |  |  |
| Fusion | 6.5 (2.4) | 7.2 (1.6) | 0.22 |
| Non-fusion | 6.8 (2.2) | 7.0 (2.3) | 0.68 |

Table S2. One-month follow-up: shared decision-making in pain treatment and satisfaction with the result of pain management.

| **Month 1** | **Group** | **Cross-section** | **n** | **Mean** | **SD** | ***p***  **Usual care vs Intervention** |
| --- | --- | --- | --- | --- | --- | --- |
| Shared decision-making | Fusion | Usual care | 30 | 5.4 | 3.6 | **0.050** |
|  |  | Intervention | 36 | 3.7 | 3.4 |  |
|  | Non-fusion | Usual care | 68 | 5.1 | 3.8 | 0.177 |
|  |  | Intervention | 38 | 4.1 | 3.9 |  |
|  | Whole group | Usual care | 98 | 5.2 | 3.7 | **0.024** |
|  |  | Intervention | 75 | 3.9 | 3.7 |  |
| Satisfaction | Fusion | Usual care | 30 | 7.2 | 2.6 | 0.402 |
|  |  | Intervention | 37 | 6.6 | 2.9 |  |
|  | Non-fusion | Usual care | 70 | 7.4 | 2.8 | 0.324 |
|  |  | Intervention | 40 | 6.9 | 2.8 |  |
|  | Whole group | Usual care | 100 | 7.4 | 2.8 | 0.150 |
|  |  | Intervention | 78 | 6.7 | 2.8 |  |

Table S3. Day 1 and one-month follow-up: pain intensity.

| **Pain intensity** | **Group** | **Cross-section** | **n** | **Mean**  **NRS** | **SD** | ***p***  **Usual care vs Intervention** |
| --- | --- | --- | --- | --- | --- | --- |
| Day 1 | Fusion | Usual care | 26 | 7.5 | 2.0 | 0.605 |
|  |  | Intervention | 32 | 7.2 | 2.6 |  |
| Month 1 |  | Usual care | 30 | 5.9 | 2.7 | 0.475 |
|  |  | Intervention | 36 | 5.4 | 3.0 |  |
| Day 1 | Non-fusion | Usual care | 73 | 5.6 | 2.6 | 0.662 |
|  |  | Intervention | 33 | 5.8 | 2.7 |  |
| Month 1 |  | Usual care | 71 | 5.0 | 2.8 | 0.455 |
|  |  | Intervention | 40 | 5.4 | 2.8 |  |
| Day 1 | Whole group | Usual care | 99 | 6.1 | 2.6 | 0.313 |
|  |  | Intervention | 66 | 6.5 | 2.7 |  |
| Month 1 |  | Usual care | 101 | 5.2 | 2.8 | 0.705 |
|  |  | Intervention | 77 | 5.4 | 2.9 |  |
